# Defining the Ideal Breast Reconstruction Procedure After Mastectomy From the Patient Perspective: A Retrospective Analysis

**DOI:** 10.1177/11782234221089597

**Published:** 2022-04-19

**Authors:** Ilias G Petrou, Céline Thomet, Omid Jamei, Ali Modarressi, Daniel F Kalbermatten, Brigitte Pittet-Cuénod

**Affiliations:** Division of Plastic, Reconstructive and Esthetic Surgery, Geneva University Hospitals and Faculty of Medicine, University of Geneva, Geneva, Switzerland

**Keywords:** Breast cancer, breast reconstruction, implant-based reconstruction, latissimus dorsi flap, deep inferior epigastric perforator, autologous breast reconstruction

## Abstract

**Background:**

An increasing number of breast cancer patients undergo immediate or secondary breast reconstruction, but the ideal method in terms of patient satisfaction remains ambiguous. We compared the 3 most common breast reconstruction techniques to determine patient satisfaction and objective outcomes.

**Methods:**

Retrospective study of 184 patients with breast cancer who underwent a reconstructive procedure between 1993 and 2011 at our institution. Procedures evaluated were implant-based reconstruction (IBR) alone, latissimus dorsi (LD) flap reconstruction with/without implant, and deep inferior epigastric perforator (DIEP) free flap reconstruction. A retrospective patient satisfaction questionnaire was sent to all women. Twenty patients from each subgroup were matched to conduct a standardized objective assessment of the sensitivity of their reconstructed breast. A blinded photographic evaluation was also performed by 3 independent observers to assess the esthetic aspect and symmetry.

**Results:**

DIEP obtained significantly higher average scores regarding the esthetic outcome, immediate reconstruction impact, and overall score in the questionnaire evaluation. The IBR had the best results in the somatosensory evaluation, with DIEP scoring better than LD. DIEP received higher scores on average than LD for the criteria of size and symmetry in the esthetic evaluation. No statistically significant differences were observed between IBR and DIEP.

**Conclusions:**

Good results were reported overall for all breast reconstruction procedures, with more reserved scores for LD. The DIEP reconstruction appeared to be the most satisfactory and best experienced reconstruction method for patients, despite the complexity of the intervention. Clinicians should be encouraged to consider DIEP as the principal choice for breast reconstruction.

## Introduction

In 2018, breast cancer was the most frequent cancer affecting women and represented 24.2% of all new cancer cases reported.^1^ The global age-standardized incidence rate was calculated at 46.3% per 100 000, rising to 88.1% per 100 000 for Switzerland, with a mortality of 12.3% per 100 000.^2^ Treatment has advanced in recent years with breast conservation therapy (BCT) superseding the classical mastectomy procedures. A recent analysis of the database of the United States Surveillance, Epidemiology, and End Results Program of the National Cancer Institute reported that the BCT was used to treat 70% of patients, while the remaining 30% had a mastectomy.^3^ Although most cases are treated with BCT to avoid the stress of breast amputation. There are still several indications for mastectomy, such as large tumor-to-breast-size ratio, multicentric tumors, positive margins of tumor excision, inflammatory breast cancer, extensive malignant microcalcifications, insufficient response to neoadjuvant chemotherapy, and local recurrence following BCT.^4^ Several studies have reported contradictory data related to a trend toward initial total mastectomy. However, it seems that preoperative magnetic resonance imaging and the individual surgeon’s attitude contribute to the increased odds of undergoing this procedure.^5^

Apart from classic indications for total mastectomy, progress in genetics will certainly lead to an increasing number of women who will still undergo preventive mastectomy.^6^ According to a recent study, the rates of contralateral prophylactic mastectomy (CPM) among women with invasive breast cancer and without genetic predispositions more than tripled from 2002 to 2012, although this approach offered no significant survival benefit over BCT.^7^ Despite the higher postoperative complication rate after CPM and immediate reconstruction, the combination of decreased anxiety and improved satisfaction of women who underwent breast reconstruction might explain the increasing insistence of patients for prophylactic interventions.^8^ In everyday practice, CPM is rarely recommended for women with unilateral breast cancer. A consensus statement from the American Society of Breast Surgeons only recommends CPM for women with a unilateral breast cancer and previous radiation or carrying a BRCA1 or BRCA2 gene mutation. It could also be offered on an individual basis for unilateral breast cancer and a genetic mutation in the CHEK2/PTEN/p53/PALB2/CDH1 gene, or in selected individuals to achieve symmetry after unilateral mastectomy.^9^

Improvements in the screening and management of breast cancer have led to a decrease of mortality and an increase in life expectancy for this population,^10^ thus emphasizing the need for high quality and long-lasting breast reconstruction procedures. Furthermore, patients are increasingly aware of scientific advances and this has led to a heightened demand for immediate and secondary breast reconstruction procedures. In daily practice, the patient will discuss the different options with the reconstructive surgeon in an explicit manner to ensure that the treatment is adapted to her needs and requests. Until now, no consensus exists for a standardized attitude toward reconstructive options. Hence, more studies are needed to highlight the differences of each approach and help patients in their choice of the ideal breast reconstruction technique. Among the different procedures, the most common is implant-based reconstruction (IBR), while reconstructions by autologous tissue, such as the latissimus dorsi (LD) musculocutaneous flap with/without implant or the abdominal deep inferior epigastric perforator (DIEP) free flap are also used with satisfactory results.^11^ The aim of this study was to compare the long-term outcomes of these 3 procedures in terms of patient satisfaction, sensitivity of the reconstructed breast, and esthetic outcome in an attempt to define the ideal reconstruction approach.

## Materials and Methods

### Study population

Eligible patients included all women who underwent IBR, LD, and DIEP reconstruction procedures, including nipple areolar complex (NAC) reconstruction, at our Geneva University Hospitals (Geneva, Switzerland) between January 1, 1993, and December 31, 2013. Exclusion criteria were contralateral conservative oncological treatment, nipple-sparing mastectomy, unachieved reconstruction without NAC reconstruction, or further surgery for recurrence. All patients were contacted by mail with information about the study and an invitation to participate. Patients who accepted to participate were asked to complete a breast reconstruction-related questionnaire in French language at home. Only those who responded to the questionnaire were included in the study. All participants provided written informed consent. The study design followed the International Society for Pharmacoeconomics and Outcomes *Research*, good research practices with a retrospective data analysis.^12^ It was also approved by the the local institutional ethics committee. The research protocols were conducted and patients were treated in accordance with the tenets of the Declaration of Helsinki.

Patients were divided into 3 subgroups (IBR, LD flap reconstruction with/without implant, and DIEP free flap breast reconstruction). As patients with LD reconstruction were limited, a sampling of women among the IBR and DIEP reconstructions was made to achieve the most homogenous grouping possible to allow an evaluation of the most effective approach. Patients from each group were matched and invited by telephone for a medical consultation to conduct a standardized clinical assessment of the objective sensitivity of their reconstructed breast and NAC, as well as a standardized photographic session for a subjective esthetic evaluation by 3 independent observers.

### Data collection

Data were retrospectively collected from medical charts on a predesigned form for each patient; patient records were then scanned and the analysis continued anonymously.

Data collection included age at the time of reconstructive surgery, type of mastectomy, uni- or bilateral procedure, and radiotherapy performed before or after reconstruction. The type of reconstruction and timing (immediate/secondary) was also recorded. Data concerning the contralateral breast were also collected, with a focus on the presence and type of previous esthetic surgery or symmetrization procedures. For those who enrolled in the second part of the study, patient questionnaire scores were then included, as well as the values of the somatosensory examination and esthetic evaluation. Results were compared between the reconstructed breast and the contralateral healthy breast of the same patient, and the reconstructed breasts of each patient included in the 3 subgroups.

### Breast satisfaction score

Breast satisfaction was evaluated by a breast reconstruction questionnaire developed in French language after considering the existing literature and methods widely used at the time of the procedure. The 36-item questionnaire investigated the sensitivity, esthetics, and impact on daily life, including global satisfaction with the reconstructed breast.

### Somatosensory evaluation

**Figure 1.**
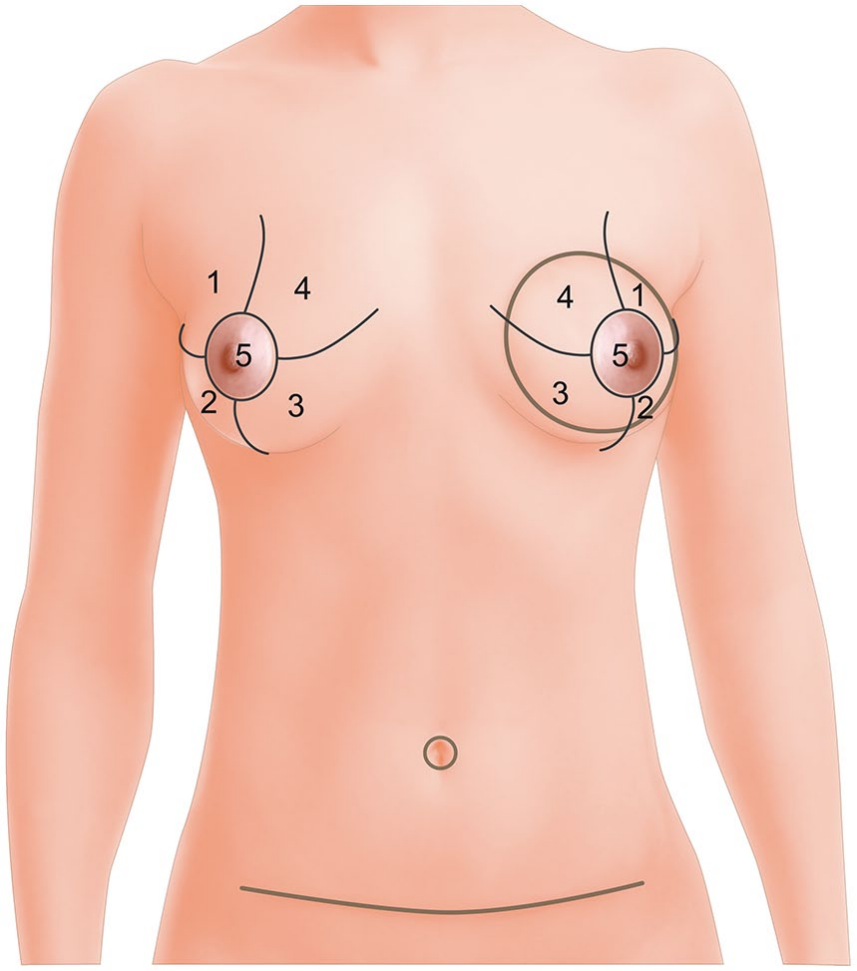
Zone definition for the somatosensory evaluation of breasts.

Objective sensitivity was measured by a somatosensory examination always performed by the same surgeon. According to a well-defined protocol, breasts were divided into 5 zones (Figure 1) and evaluated individually according to 3 sensory modalities. (1) Pressure sensitivity: determination of the quantitative threshold of pressure detection by Semmes-Weinstein monofilament examination (graded scale) with an application force ranging from 0.008 to 300 g. Monofilament values were recorded for all 5 areas of both breasts. (2) Vibration sensitivity: determination of the quantitative threshold of vibration detection by examination with an IKAR probe/vibralgic vibrostimulator (LMT, Ecublens, Switzerland) (gradual scale). The vibralgic consists of a signal generator and a vibrostimulator. The generator produces a sinusoidal signal with a frequency between 30 and 1000 Hz and a voltage between 0.1 and 4.8 V. The electrical signal is converted to a mechanical displacement by the stimulator in the same manner as in a loudspeaker. (3) Hot-cold discrimination: determination of the presence or absence of hot-cold discrimination by examination with BD vacutainer tubes (Becton Dickinson, Franklin Lakes, NJ) filled with water at 16°C (cold) or 43°C (hot). For the statistical analyses, we compared controlateral normal breasts without any reconstruction or other operation. This group was used as a reference category in multivariate models.

### Esthetic–symmetry evaluation

All women included in the objective somatosensory study were photographed in standardized frontal, lateral and oblique breast views. Blinded photographs were then presented to 3 examiners (a resident with limited exposure to plastic surgery procedures, a nurse, and a specialized plastic surgeon) who were asked to evaluate the shape, symmetry, and esthetics of the reconstructed breast, including the symmetry and position of the reconstructed NAC and nipple. Responses were measured on a 5-point Likert scale (1 = “strongly disagree” and 5 = “strongly agree”). To avoid any bias, the examiner was not aware of the patient’s questionnaire scores.

### Statistical analysis

Patient characteristics (ie, age, immediate or secondary reconstruction, radiotherapy, year of reconstruction, type of immediate reconstruction, and laterality) were described using the mean (standard deviation) or count (percentage), as appropriate. Continuous variables were compared with the independent samples *t* test or the Kruskall-Wallis test. A multiple linear regression model was used to adjust for patient characteristics and to calculate mean differences. We examined differences in patient characteristics among the 3 different types of reconstruction using the chi-square and/or Fisher exact test. Generalized equations were also used to calculate associations between the outcomes, presence of radiotherapy, and timing (immediate or secondary) of the reconstruction. Statistical differences were determined using 95% confidence intervals. The primary outcome of interest was the self-report scores of breast satisfaction following reconstruction. Secondary outcomes were an assessment of the esthetic outcome and a somatosensory evaluation. A *P* value < .05 was considered statistically significant. All statistical analyses were performed using IBM SPSS version 23.0 for Windows (IBM Corp., Armonk, NY).

## Results

### Patient characteristics

In total, 441 eligible patients were identified; 184 (43%) women responded to the questionnaire and were included in the study. Among these, 104 (56.6%) had IBR, 40 (22.2%) LD, and 40 (22.2%) had DIEP reconstruction. In the IBR group, only 15.2% of patients had radiotherapy (pre- and post-reconstruction) versus 71.8% and 60.0% in the LD and DIEP groups, respectively. Immediate reconstruction was performed in 84.8% of IBR patients, 59% in the LD group, and approximately 30% in the DIEP group. There were significantly more secondary reconstructions in the DIEP and LD groups, but the proportions of radiotherapy were not significantly different between the 2 groups. Mean differences in patient age between the time of the study and time of reconstruction were 4.3 years for DIEP, 7.3 for LD, and 7.5 for IBR (Table 1).

**Table 1.** Patient characteristics.

		DIEP (n = 40)		LD (n = 40)		IBR (n = 104)		Global *P* value	Test	DIEP vs LD	DIEP vs IBR	IBR vs LD
Radiotherapy (Rx)	No	18	45%	12	30%	88	85%	<.001	Chi2	0.166	0.000	0.000
	Yes	22	55%	28	70%	16	15%					
Immediate	Immediate	13	32.5%	21	52.5%	86	82.7%	.000	Chi2	0.043	0.000	0.001
	Secondary	27	67.5%	17	42.5%	18	17.3%					
	Missing Data	0		2		0						
Radiotherapy (Rx)*	Without Rx, prim.	9	22.5%	6	15%	74	71.1%	.000	Fisher	0.025	0.000	0.000
Immediate/Secondary	Without Rx, sec.	9	22.5%	6	15%	14	13.5%					
	With Rx, prim.	4	10%	15	37.5%	12	11.5%					
	With Rx, sec.	18	45%	11	27.5%	4	3.9%					
Age 1, y (time of the study)	Median (min—max)	55.3	36.6 to 71.8	56.4	41.9 to 73.2	60.5	19.1 to 80.9	.005	Kruskall-Wallis	0.953	0.009	0.010
Age 2, y (time of the reconstruction)	Median (min—max)	51.0	33.7 to 67.0	49.1	30.5 to 69.2	53.0	17.6 to 77.1	.12	Kruskall-Wallis	0.32	0.34	0.05
Delay between surgery and mastectomy, y	Mean	1.78	(0 to 12.44)	1.50	0 to 11.96	0.69	0 to 20.01	.000	Kruskall-Wallis	0.08	<0.001	0.0009

Abbreviations: DIEP, deep inferior epigastric perforator; IBR, implant-based reconstruction; LD, latissimus dorsi.

### Summary of breast satisfaction questionnaire data

#### Comparison of overall scores

**Figure 2.**
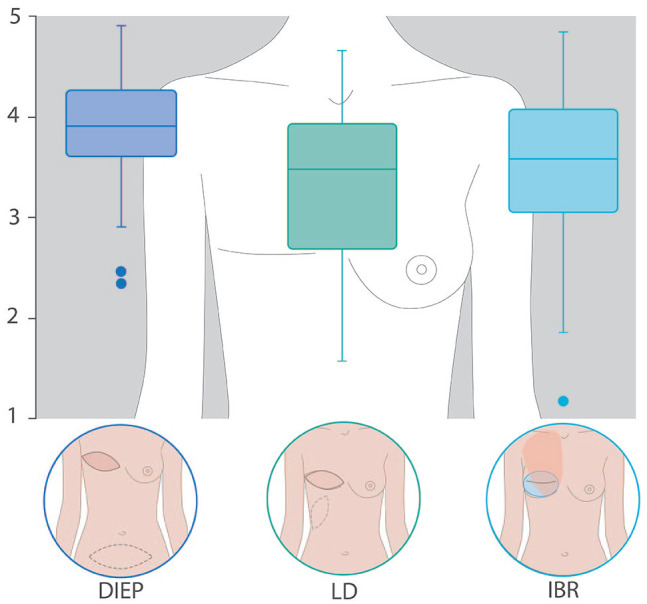
Overall score—questionnaire results by group. DIEP indicates deep inferior epigastric perforator; IBR, implant-based reconstruction; LD, latissimus dorsi.

**Figure 3.**
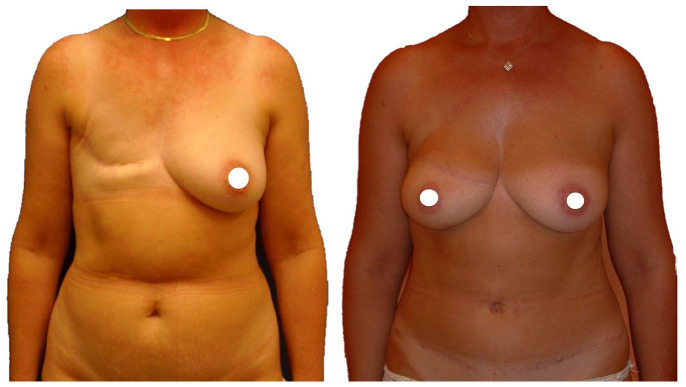
DIEP breast reconstruction: before–after result after 6 years postoperative. DIEP indicates deep inferior epigastric perforator.

In univariate analysis, we observed that DIEP obtained a mean sensitivity score significantly higher than LD, but not significantly higher than IBR. DIEP also obtained significantly higher average scores than LD and IBR for the esthetics and immediate reconstruction impact (but at the limit of significance compared with IBR [*P* = .07]), including the overall score (Figure 2). An analysis of each question also showed that DIEP (Figure 3) had significantly better scores for pain, natural feeling and symmetry than LD and IBR (Table 2).

**Table 2.** Questionnaire results by group.

	Mean (SE: standard error)						*P* value			
	DIEP	SE	LD	SE	IBR	SE	Overall *P* value	DIEP vs LD	DIEP vs IBR	LD vs IBR
Sensitivity	3.34	0.11	2.95	0.10	3.19	0.07	.04	**0.02**	0.32	**0.04**
Esthetics	4.11	0.11	3.51	0.16	3.55	0.10	.0059	**0.0081**	**0.0025**	0.93
Secondary reconstruction impact	3.04	0.17	2.47	0.36	2.64	0.26	.42	0.23	0.37	0.69
Immediate reconstruction impact	4.10	0.15	3.48	0.20	3.74	0.11	.046	**0.01**	**0.07**	0.27
Aggregation of differences	1.59	0.23	1.41	0.35	1.48	0.29	.999	0.98	0.92	0.96
Overall score	3.90	0.10	3.33	0.13	3.50	0.08	.0031	**0.0019**	**0.0036**	0.35

Abbreviations: DIEP, deep inferior epigastric perforator; IBR, implant-based reconstruction; LD, latissimus dorsi; SE, standard error.

#### Comparison of radiation therapy and immediate/secondary reconstruction scores

In patients with DIEP reconstruction, mean scores were higher for women who did not have radiation therapy compared with those who did, but there were no significant differences between the 2 groups. However, numbers were small and we noted that the immediate impact was at the limit of significance (*P* = .05). Secondary reconstructions in this group appeared to have higher mean scores than IBR patients, but this was not statistically significant (Table S1). When taking into account confounding factors (radiotherapy, immediate/secondary reconstruction, age), mean scores for DIEP were significantly higher than LD for sensitivity, esthetics and immediate impact, including the overall score. DIEP also scored significantly higher than IBR for esthetics and on the overall score (Table S2).

There was no evidence of a different group effect between levels of radiotherapy. By contrast, we found an effect of the timing of reconstruction between immediate and secondary reconstruction (Table S3). In patients with secondary reconstruction, those who underwent DIEP had significantly higher esthetic scores and overall scores than those with LD reconstruction. Of note, although women with DIEP reconstruction had a significantly higher esthetic score and overall score than IBR patients, these differences were not observed in the group who had immediate reconstruction (Table S4).

### Somatosensory evaluation

The IBR group was significantly better than DIEP for sensitivity to heat, vibrations, and monofilament. Results for sensitivity to cold were similar, but at the limit of significance (*P* = .09). The IBR group scored significantly better than LD for sensitivity to hot, cold, and monofilament, but without any significant difference for sensitivity to vibrations. Heat or cold sensitivity of breasts reconstructed by DIEP appeared to be better than for LD, but the results were not significant. Sensitivity to vibrations of breasts reconstructed by DIEP scored less than those reconstructed by LD. Among breasts reconstructed by the DIEP or LD technique, no significant effect of radiotherapy was observed on the different sensitivities (Table 3).

**Table 3.** Somatosensory evaluation.

Comparison	Sensitivity to cold			Sensitivity to heat			Vibration			Monofilament		
	Mean diff.	95% CI	*P* value	Mean diff.	95% CI	*P* value	Mean diff.	95% CI	*P* value	Mean diff.	95% CI	*P* value
DIEP vs IBR	−1.4	−3.0 to 0.2	.09	−1.6	−3.1 to −0.04	.04	0.08	0.008 to 0.152	.03	73.2	27.8 to 118.5	.002
DIEP vs LD	1.1	−0.6 to 2.8	.2	1.5	−0.07 to 3.2	.06	0.10	0.02 to 0.17	.009	10.9	−37.2 to 58.9	.7
LD vs IBR	−2.5	−4.1 to −0.9	.003	−3.1	−4.7 to −1.6	<.001	−0.02	−0.09 to 0.06	.7	62.3	16.8 to 107.9	.007

Abbreviations: CI, confidence interval; DIEP, deep inferior epigastric perforator; IBR, implant-based reconstruction; LD, latissimus dorsi.

### Esthetic evaluation

An evaluation of photographs showed that the DIEP technique received on average higher scores than LD for the criteria of size and symmetry (average difference between the 2 techniques of approximately 1 point on the Likert scale). The DIEP technique had also higher overall scores than LD for the esthetic criterion (+0.7 points on average; *P* = .06), but the difference was not statistically significant. No statistically significant differences were observed between IBR and DIEP. No significant association was observed between the type of reconstruction and the scores assessed on photographs for other items, such as symmetrical NAC or NAC position, symmetrical nipples and color (Table 4).

**Table 4.** Subjective photographic comparison between IBR, LD and DIEP reconstruction techniques.

	Difference	95% CI	95% CI	*P* value	Overall *P* value
Size					
DIEP vs IBR	0.5	−0.4	1.3	.29	.052
**DIEP vs LD**	**0.9**	**0.2**	**1.7**	.**01**	
LD vs IBR	−0.5	−1.4	0.4	.29	
Symmetry					.048
DIEP vs IBR	0.4	−0.5	1.4	.36	
**DIEP vs LD**	**1.0**	**0.2**	**1.9**	.**01**	
LD vs IBR	−0.6	−1.6	0.4	.22	
Esthetic					.17
DIEP vs IBR	0.2	−0.7	1.1	.61	
DIEP vs LD	0.7	0.0	1.5	.06	
LD vs IBR	−0.5	−1.4	0.4	.27	
Symmetrical NAC					.68
DIEP vs IBR	0.1	−0.8	1.0	.87	
DIEP vs LD	−0.3	−1.1	0.5	.47	
LD vs IBR	0.4	−0.6	1.3	.44	
Symmetrical NAC position					.13
DIEP vs IBR	0.8	−0.1	1.7	.09	
DIEP vs LD	0.7	−0.1	1.6	.09	
LD vs IBR	0.1	−0.9	1.0	.87	
Symmetrical nipples					.13
DIEP vs IBR	−0.2	−0.8	0.5	.65	
DIEP vs LD	−0.3	−0.9	0.3	.27	
LD vs IBR	0.2	−0.5	0.9	.62	
Areola color					.61
DIEP vs IBR	−0.5	−1.4	0.5	.33	
DIEP vs LD	−0.1	−0.9	0.8	.87	
LD vs IBR	−0.4	−1.4	0.6	.43	
Nipple color					.16
DIEP vs IBR	−0.7	−1.7	0.2	.13	
DIEP vs LD	−0.7	−1.5	0.1	.10	
LD vs IBR	0.0	−1.0	0.9	.95	

Abbreviations: CI, confidence interval; DIEP, deep inferior epigastric perforator; IBR, implant-based reconstruction; LD, latissimus dorsi; NAC, nipple areolar complex.

## Discussion

In our study, DIEP breast reconstruction appeared to give the best results in terms of patient satisfaction, despite the fact that it is a burdensome procedure with a considerable donor site scar (Figure 3). In addition, included patients were mainly irradiated and benefited from secondary reconstructions with sizable skin demands. In theory, this type of operation should therefore score even better in the absence of radiation therapy. Furthermore, DIEP has been validated as a safe and efficient salvage procedure after IBR failure.^13^ Although IBR reconstruction appears to score better in terms of sensation, recent advancements in the DIEP technique with neurotization through the intercostal nerves offer solid promises for a satisfactory sensation recovery.^14,15^ LD reconstruction with/without implant was associated with the worst scores in terms of patient satisfaction, sensitivity, and subjective esthetic results. This could be explained by the substantial need for skin and soft tissue reconstruction or the addition of an implant to gain more volume, thus resulting in inferior outcomes. Moreover, LD reconstruction is sometimes implemented as a salvage procedure and outcomes could lean toward worst scores due to previous reconstructive failures and a natural fatigue in their “journey” to achieve a satisfactory appearance. However, recent adjustments and refinements in the LD reconstruction technique have been proposed with emphasis on patient selection to achieve the best possible outcomes.^16^

A multidisciplinary, pre-therapeutic consultation was set up at our hospital in 2000 for patients diagnosed with breast cancer and very rapidly increased the number of women benefiting from breast reconstruction, which is now systematically offered. As expected, this increase in activity boosted the number of immediate reconstructions. Among these, IBR is the most common procedure, but an increasing number of autologous reconstructions by LD or DIEP are now also offered. Moreover, our technique of immediate IBR with a permanent single-step implant results in a satisfactory outcome by avoiding the multiple steps of expander implementation^17^ and this may explain why there is a clear majority of patients with immediate IBR in our cohort.

IBR can result in good outcomes in well-selected cases in the absence of irradiation.^18^ Although we report only direct-to-implant retro-pectoral IBR, subcutaneous placement of implants is gaining popularity with satisfactory cosmetic long-term results.^19^ However, in recent years, it became obvious that adjuvant radiotherapy was clearly linked to increased complication rates when combined with IBR.^20,21^ As a result, we prefer autologous secondary procedure techniques for patients who have already undergone radiation therapy. In our experience, the “workhorse” flaps for breast reconstruction are the LD when a pedicled flap is needed, and DIEP as a free tissue transfer.

Autologous reconstruction methods such as LD or DIEP have shown higher patient-reported satisfaction rates and lower reconstruction failure rates, particularly when performed after radiotherapy.^13^ Although these methods have been reserved traditionally for secondary reconstruction, there seems to be a clear advantage of immediate procedures in long-term patient-reported outcomes.^22^ We showed a superiority of DIEP over the LD approach, which should be always considered as a salvage procedure. Indeed, autologous flap breast reconstruction seems to be in gaining pace in the quest to identify the ideal reconstruction technique. However, it has been observed that the flap-related complications, especially in immediate reconstructions, can provoke more emotional stress and are associated with lower satisfaction rates than any other procedure.^23^

To our knowledge, there is a limited literature comparing these 3 most common breast reconstructive techniques. The inclusion of sensory and esthetic subjective outcomes also provides an added value to this study. Furthermore, the median long follow-up periods between the time of reconstruction and time of study strengthen the importance of our outcomes. Our data are coherent with the existing literature, but provide additional findings in the differences and expected outcomes. The main significance is that surgeons should extensively discuss the risks and benefits of each technique and should favor DIEP over the LD flap if possible when an autologous procedure is required. Patients should be thoroughly informed about the realistic expectations and potential complications when they make choices about breast cancer surgery. A potential reconstructive failure must always be addressed during preoperative consultations, as well as alternative options if an adverse event occurs. Surgeons should be cautious and meticulous when it comes to patient and procedure selection to achieve the best possible outcomes. Patient emotional well-being and satisfaction rates are of great significance when attempting to identify the ideal reconstructed breast.

Nevertheless, some limitations of our study must be addressed. First, we acknowledge the small sample size due to the poor participation of patients (43%). Second, there are some differences in the patient groups, particularly between IBR and LD or DIEP, as autologous procedures were implemented more on irradiated patients. Third, this is a retrospective study. Fourth, our findings may reflect lead-time bias as patients who underwent autologous reconstruction were interviewed at a different time from reconstruction compared to IBR procedures. As IBR is mostly immediate, patients were closer to their initial operation than the autologous groups, which are composed of more secondary procedures. Importantly, the immediate group never had to live a period of their life without their breast. Thus, it is possible that patients who overcame a period without their natural breast evaluated differently reconstruction outcomes. Finally, given our recruitment method, our findings may reflect women who successfully completed their desired reconstructive technique and may not be applicable to subgroups of women who did not achieve this objective.

We report overall good results for all breast reconstruction procedures, but with more reserved scores for LD reconstruction. Interpretation of the relatively high satisfaction rate of IBR should take into account the particularly low rate of adjuvant radiotherapy in this group compared to autologous reconstructions. DIEP reconstruction appears to be the most satisfactory and best experienced reconstruction method from the patient perspective, despite the complexity of the intervention. Clinicians should be encouraged to consider DIEP as a principal choice for breast reconstruction.

## Supplemental Material

sj-docx-1-bcb-10.1177_11782234221089597 – Supplemental material for Defining the Ideal Breast Reconstruction Procedure After Mastectomy From the Patient Perspective: A Retrospective Analysis Click here for additional data file. Supplemental material, sj-docx-1-bcb-10.1177_11782234221089597 for Defining the Ideal Breast Reconstruction Procedure After Mastectomy From the Patient Perspective: A Retrospective Analysis by Ilias G Petrou, Céline Thomet, Omid Jamei, Ali Modarressi, Daniel F Kalbermatten and Brigitte Pittet-Cuénod in Breast Cancer: Basic and Clinical Research
