# Supplementary material for: Defining the Ideal Breast Reconstruction Procedure After Mastectomy From the Patient Perspective: A Retrospective Analysis
Source: Breast Cancer (Auckl). 2022 Apr 19;16:11782234221089597. doi: 10.1177/11782234221089597 (PMC9021510; doi:10.1177/11782234221089597)
Supplement: sj-docx-1-bcb-10.1177_11782234221089597 – Supplemental material for Defining the Ideal Breast Reconstruction Procedure After Mastectomy From the Patient Perspective: A Retrospective Analysis [file sj-docx-1-bcb-10.1177_11782234221089597.docx]

**Supplementary Tables**

**Table S1**. Comparison of radiotherapy and immediate and secondary reconstruction scores.

| **DIEP** | | **No Radiotherapy** | | | | | | | | | **Radiotherapy** | | | | | | | | |  | | | | **Immediate** | | | | | | | | | **Secondary** | | | | | | |  | | |
| --- | --- | --- | --- | --- | --- | --- | --- | --- | --- | --- | --- | --- | --- | --- | --- | --- | --- | --- | --- | --- | --- | --- | --- | --- | --- | --- | --- | --- | --- | --- | --- | --- | --- | --- | --- | --- | --- | --- | --- | --- | --- | --- |
|  | | N | | | Mean | | | Standard error | | | N | | | Mean | | | Standard error | | | p value | | | | N | Mean | | | Standard error | | | | | N | Mean | | | Standard error | | | p value | | |
| **Sensitivity** | | 18 | | | 3.41 | | | 0.18 | | | 22 | | | 3.28 | | | 0.15 | | | 0.6046 | | | | 13 | 3.11 | | | 0.16 | | | | | 27 | 3.45 | | | 0.15 | | | 0.1472 | | |
| **Aesthetics** | | 18 | | | 4.22 | | | 0.16 | | | 22 | | | 4.01 | | | 0.15 | | | 0.3006 | | | | 13 | 3.91 | | | 0.18 | | | | | 27 | 4.20 | | | 0.15 | | | 0.1321 | | |
| **Immediate impact** | | 18 | | | 4.42 | | | 0.20 | | | 22 | | | 3.84 | | | 0.21 | | | 0.0533 | | | | 13 | 3.82 | | | 0.30 | | | | | 27 | 4.24 | | | 0.21 | | | 0.1725 | | |
| **Overall score** | | 18 | | | 4.00 | | | 0.16 | | | 22 | | | 3.81 | | | 0.12 | | | 0.2706 | | | | 13 | 3.62 | | | 0.16 | | | | | 27 | 4.03 | | | 0.12 | | | 0.1117 | | |
| **Secondary impact** | | 8 | | | 3.47 | | | 0.28 | | | 16 | | | 2.83 | | | 0.20 | | | 0.1658 | | | |  |  | | |  | | | | | 24 | 3.00 | | | 0.17 | | |  | | |
| **Aggregation from differences** | | 8 | | | 2.22 | | | 0.48 | | | 17 | | | 1.29 | | | 0.24 | | | 0.1065 | | | |  |  | | |  | | | | | 25 | 1.60 | | | 0.23 | | |  | | |
| **LD** | | **No radiotherapy** | | | | | | | | **Radiotherapy** | | | | | | | | |  | | **Immediate** | | | | | | | | | **Secondary** | | | | | | | | |  | | |  |
|  | | N | | Mean | | | Standard error | | | N | | | Mean | | | Standard error | | | p value | | N | | Mean | | | | Standard error | | | N | | Mean | | | | Standard error | | | p value | | |  |
| **Sensitivity** | | 12 | | 2.82 | | | 0.19 | | | 28 | | | 3.01 | | | 0.12 | | | 0.2670 | | 21 | | 2.94 | | | | 0.14 | | | 17 | | 2.91 | | | | 0.15 | | | 0.7018 | | |  |
| **Aesthetics** | | 12 | | 3.29 | | | 0.30 | | | 28 | | | 3.60 | | | 0.19 | | | 0.2745 | | 21 | | 3.80 | | | | 0.14 | | | 17 | | 3.09 | | | | 0.31 | | | 0.1061 | | |  |
| **Immediate impact** | | 12 | | 3.22 | | | 0.37 | | | 28 | | | 3.59 | | | 0.24 | | | 0.3585 | | 21 | | 3.62 | | | | 0.25 | | | 17 | | 3.16 | | | | 0.33 | | | 0.2446 | | |  |
| **Overall score** | | 12 | | 3.20 | | | 0.24 | | | 26 | | | 3.40 | | | 0.16 | | | 0.4142 | | 21 | | 3.51 | | | | 0.14 | | | 17 | | 3.12 | | | | 0.23 | | | 0.2231 | | |  |
| **Secondary impact** | | 4 | | 2.31 | | | 0.75 | | | 8 | | | 2.55 | | | 0.44 | | | 0.9311 | |  | |  | | | |  | | | 12 | | 2.47 | | | | 0.36 | | |  | | |  |
| **Aggregation from differences** | | 5 | | 1.45 | | | 0.65 | | | 9 | | | 1.39 | | | 0.45 | | | 0.9463 | |  | |  | | | |  | | | 14 | | 1.41 | | | | 0.35 | | |  | | |  |
| **IBR** | **No radiotherapy** | | | | | | | | **Radiotherapy** | | | | | | | | |  | | | **Immediate** | | | | | | | | **Secondary** | | | | | | | | |  | | |  |  |
|  | N | | Mean | | | Standard error | | | N | | | Mean | | | Standard error | | | p value | | | N | Mean | | | | Standard error | | | N | | Mean | | | | Standard error | | | p value | | |  |  |
| **Sensitivity** | 88 | | 3.19 | | | 0.07 | | | 16 | | | 3.19 | | | 0.18 | | | 0.8604 | | | 86 | 3.21 | | | | 0.08 | | | 18 | | 3.11 | | | | 0.13 | | | 0.8130 | | |  |  |
| **Aesthetics** | 87 | | 3.60 | | | 0.10 | | | 16 | | | 3.31 | | | 0.26 | | | 0.2587 | | | 85 | 3.60 | | | | 0.11 | | | 18 | | 3.33 | | | | 0.21 | | | 0.2289 | | |  |  |
| **Immediate impact** | 88 | | 3.72 | | | 0.12 | | | 16 | | | 3.85 | | | 0.27 | | | 0.6904 | | | 86 | 3.72 | | | | 0.13 | | | 18 | | 3.84 | | | | 0.22 | | | 0.9966 | | |  |  |
| **Overall score** | 88 | | 3.51 | | | 0.08 | | | 16 | | | 3.42 | | | 0.20 | | | 0.7593 | | | 86 | 3.52 | | | | 0.09 | | | 18 | | 3.42 | | | | 0.15 | | | 0.6001 | | |  |  |
| **Secondary impact** | 11 | | 2.68 | | | 0.32 | | | 4 | | | 2.53 | | | 0.45 | | | 0.5547 | | |  |  | | | |  | | | 15 | | 2.64 | | | | 0.26 | | |  | | |  |  |
| **Aggregation from differences** | 11 | | 1.50 | | | 0.35 | | | 4 | | | 1.44 | | | 0.53 | | | 0.9477 | | |  |  | | | |  | | | 15 | | 1.48 | | | | 0.29 | | |  | | |  |  |

DIEP, deep inferior epigastric perforator; LD, latissimus dorsi; IBR, implant-based reconstruction

**Table S2**. Comparison taking into account the confounding factors (radiotherapy, immediate/secondary timing of reconstruction, age).

|  | **Sensitivity** | | | **Esthetics** | | | | **Immediate impact** | | | **Overall score** | | | | **Secondary impact** | | | | **Aggregation from differences** | | | |  |
| --- | --- | --- | --- | --- | --- | --- | --- | --- | --- | --- | --- | --- | --- | --- | --- | --- | --- | --- | --- | --- | --- | --- | --- |
| **Comparison** | **Mean**  **diff.** | **SE** | **p**  **value** | | **Mean**  **diff.** | **SE** | **p**  **value** | **Mean**  **diff** | **SE** | **p**  **value** | | **Mean  diff** | **SE** | **p value** | | **Mean  diff** | **SE** | **p value** | | **Mean**  **diff** | **SE** | **p value** | |
| **LD minus DIEP** | -0.41 | 0.15 | 0.009 | | -0.67 | 0.22 | 0.002 | -0.67 | 0.26 | 0.011 | | -0.57 | 0.17 | 0.001 | | -0.5 | 0.35 | 0.15 | | -0.11 | 0.39 | 0.78 | |
| **IBR minus DIEP** | -0.24 | 0.15 | 0.107 | | -0.76 | 0.21 | <0.0001 | -0.49 | 0.25 | 0.05 | | -0.53 | 0.16 | 0.001 | | -0.7 | 0.35 | 0.05 | | -0.48 | 0.42 | 0.26 | |
| **IBR minus LD** | 0.41 | 0.15 | 0.009 | | -0.09 | 0.21 | 0.65 | 0.18 | 0.25 | 0.47 | | 0.04 | 0.16 | 0.81 | | -0.19 | 0.4 | 0.64 | | -0.37 | 0.47 | 0.43 | |
| **overall p value** | 0.0075 |  |  | | 0.009 |  |  | 0.06 |  |  | | 0.0037 |  |  | | 0.06 |  |  | | 0.3 |  |  | |

*SE= standard error

DIEP, deep inferior epigastric perforator; LD, latissimus dorsi; IBR, implant-based reconstruction

**Table S3**. Radiotherapy and immediate/secondary reconstruction effects.

|  | **p value for the interaction between groups** | |
| --- | --- | --- |
|  | **Radiotherapy** | **Immediate/secondary** |
| **Sensitivity** | 0.65 | 0.3 |
| **Aesthetics** | 0.46 | 0.06 |
| **Immediate impact** | 0.19 | 0.21 |
| **Overall score** | 0.55 | 0.06 |
| **Aggregation from differences** | 0.6 |  |
| **Secondary impact** | 0.57 |  |

**Table S4.** Aesthetics and overall score

|  | **Aesthetics** | | | **Overall score** | | |
| --- | --- | --- | --- | --- | --- | --- |
| **Comparison** | **Mean**  **diff.** | **SE** | **p**  **value** | **Mean  diff** | **SE** | **p value** |
| **LD minus DIEP** | -0.15 | 0.34 | 0.66 | -0.15 | 0.28 | 0.59 |
| **IBR minus DIEP** | -0.37 | 0.28 | 0.20 | -0.21 | 0.23 | 0.37 |
| **IBR minus LD** | -0.22 | 0.26 | 0.42 | -0.06 | 0.22 | 0.78 |
| **overall p value** | 0.02 |  |  | 0.66 |  |  |

**Adjusted mean differences for immediate reconstruction**

|  | **Aesthetics** | | | **Overall score** | | |
| --- | --- | --- | --- | --- | --- | --- |
| **Comparison** | **Mean**  **diff.** | **SE** | **p**  **value** | **Mean  diff** | **SE** | **p value** |
| **LD minus DIEP** | -1.11 | 0.29 | 0.00 | -0.90 | 0.22 | 0.00 |
| **IBR minus DIEP** | -1.09 | 0.32 | 0.00 | -0.79 | 0.24 | 0.00 |
| **IBR minus LD** | 0.02 | 0.35 | 0.95 | 0.11 | 0.26 | 0.68 |
| **overall p value** | <0.001 |  |  | <0.001 |  |  |

**Adjusted mean differences for secondary reconstruction**

SE, standard error.

DIEP, deep inferior epigastric perforator; LD, latissimus dorsi; IBR, implant-based reconstruction
